# SeAMotE: a method for high-throughput motif discovery in nucleic acid sequences

**DOI:** 10.1186/1471-2164-15-925

**Published:** 2014-10-23

**Authors:** Federico Agostini, Davide Cirillo, Riccardo Delli Ponti, Gian Gaetano Tartaglia

**Affiliations:** Gene Function and Evolution, Centre for Genomic Regulation (CRG), C/ Dr. Aiguader 88, 08003 Barcelona, Spain; Universitat Pompeu Fabra (UPF), C/ Dr. Aiguader 88, 08003 Barcelona, Spain; Institució Catalana de Recerca i Estudis Avançats (ICREA), 23 Passeig Lluís Companys, 08010 Barcelona, Spain

**Keywords:** Discriminative motif discovery, Nucleic acids, ChIP-seq, CLIP-seq

## Abstract

**Background:**

The large amount of data produced by high-throughput sequencing poses new computational challenges. In the last decade, several tools have been developed for the identification of transcription and splicing factor binding sites.

**Results:**

Here, we introduce the SeAMotE (Sequence Analysis of Motifs Enrichment) algorithm for discovery of regulatory regions in nucleic acid sequences. SeAMotE provides (i) a robust analysis of high-throughput sequence sets, (ii) a motif search based on pattern occurrences and (iii) an easy-to-use web-server interface. We applied our method to recently published data including 351 chromatin immunoprecipitation (ChIP) and 13 crosslinking immunoprecipitation (CLIP) experiments and compared our results with those of other well-established motif discovery tools. SeAMotE shows an average accuracy of 80% in finding discriminative motifs and outperforms other methods available in literature.

**Conclusions:**

SeAMotE is a fast, accurate and flexible algorithm for the identification of sequence patterns involved in protein-DNA and protein-RNA recognition. The server can be freely accessed at http://s.tartaglialab.com/new_submission/seamote.

**Electronic supplementary material:**

The online version of this article (doi:10.1186/1471-2164-15-925) contains supplementary material, which is available to authorized users.

## Background

Transcriptional and post-transcriptional events involve the interplay between protein effectors and nucleic acid targets, whose physical interaction is guided by sequence motifs and specific structural elements [1–3]. Motifs are usually defined as short nucleotide sequence patterns of length *k* (*k*-mers) and represented with matrices containing the probabilities to find nucleotides in specific positions (position weighted matrices PWMs). In the past decade, the advancement of high-throughput technologies contributed to the generation of a large amount of genomic data [4], promoting development of computational methods to detect regulatory elements such as transcription and splicing factor binding sites [5]. One fundamental requirement of methods for large-scale analysis is that relevant features (e.g., recognition motifs) are identified with good accuracy and in reasonable time [6, 7]. Very importantly, algorithms should be as comprehensive as possible to provide insights into the nature of regulatory elements in their real genomic context, which requires analysis of different biological sets [8].

As discussed by Ma *et al.* [9] and Weirauch *et al.* [10], there are several algorithms for *de novo* motif discovery, but only few are capable of performing a discriminative analysis (i.e., comparison between two sets) on high-throughput datasets:

- ● DREME [11] restricts the search for sequence motifs to a simplified form of “regular expression” (RE) words over the IUPAC alphabet, which consists of 11 wildcard characters in addition to the standard DNA alphabet (ACGT). To save computation time, DREME estimates the significance of RE candidates by a heuristic search without scanning the whole input sequences [11];
- ● CMF (Contrast Motif Finder) [12] is designed to discriminate between two sets of DNA sequences through non-discrete PWMs. The method takes into account false positive sites when updating PWMs and related variables;
- ● DECOD (DECOnvolved Discriminative motif discovery) [13] uses *k*-mer counts. To compensate for errors introduced from ignoring the context of the *k*-mer, DECOD uses a deconvolution method that accounts for the higher rates of *k*-mers containing subsets of the true motif;
- ● XXmotif (eXhaustive, weight matriX-based motif discovery) [14] consists of i) a masking stage, where repeat regions, compositionally biased segments and homologous segment pairs are identified; ii) a pattern stage, where p-value enrichments are calculated for seed patterns using all 5-mers (with up to two degenerate IUPAC characters); iii) and a PWM stage, where thousands of candidate PWMs are merged.

Despite the variety of motif discrimination approaches, knowledge of programming languages [8, 15] and acquaintance with web-based bioinformatics platforms [7, 16] often limit their use among non-specialists.

In this article, we introduce SeAMotE, a web-server to perform *de novo* discriminative motif discovery in nucleic acid datasets. We present an approach that enables the exhaustive search of distinctive patterns in large sets of sequences, in a reasonable amount of computational time and with an easy-to-use user interface.

## Methods

**Figure 1:**
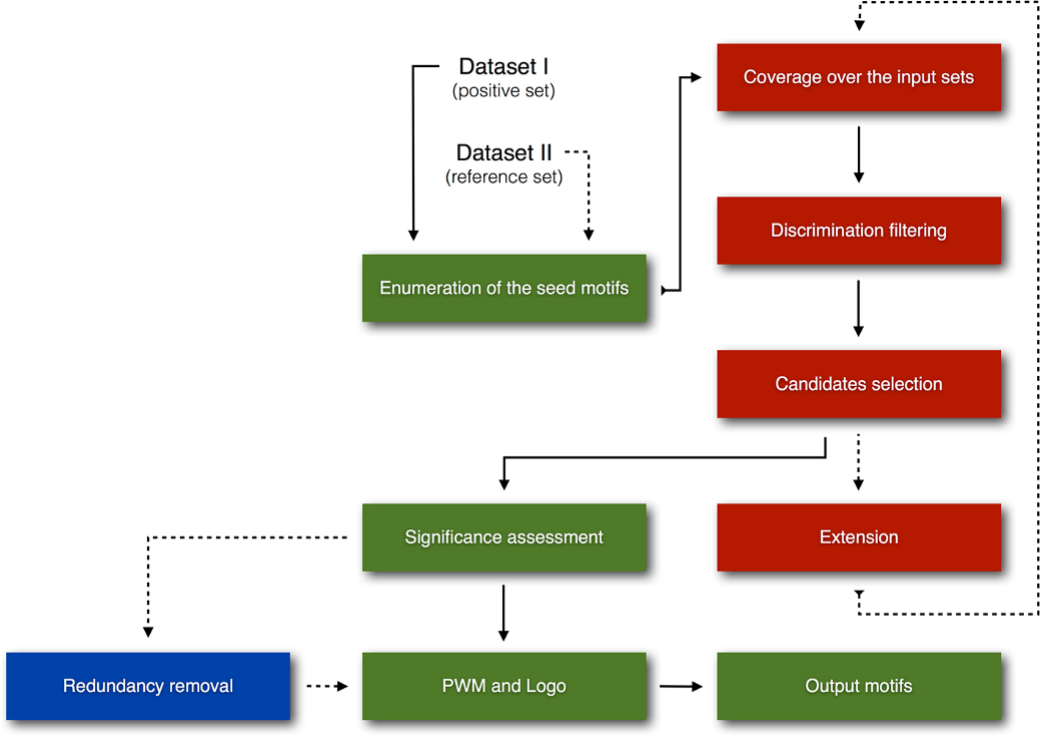
**SeAMotE workflow.** Illustration of the method pipeline: red boxes indicate the coverage calculation and seed extension loop; dashed arrows and the blue box represent conditional steps that depend on the user-definable variables, such as providing or selecting a specific background set or filtering out patterns that are closely related.

SeAMotE is based on the generation of a pool of nucleotide seeds followed by “zero or one occurrence per sequence” (ZOOPs) model testing [17] coupled with pattern extension and refinement [8]. SeAMotE includes a number of unique features that make the algorithm simple to run and very accurate. The user can i) set a coverage threshold that is employed in the selection of enriched motifs in the positive set (foreground), ii) choose among multiple reference (background) set options and iii) include a redundancy removal step to increase the variability of discovered motifs. As shown in Figure 1, SeAMotE workflow comprises a series of steps that can be summarized as follows:

1. Generation of a pool of *k*-mers seed motifs using the IUPAC alphabet;
2. Evaluation of the coverage of each pattern in the positive and reference sets;
3. Determination of enriched (Fisher’s exact test) and differentially represented (Youden’s index = Sensitivity + Specificity - 1) motifs;
4. Extension of selected seeds by adding a IUPAC letter in the *k* + 1 position;
5. Re-iteration of steps 2-4 until the enrichement of at least one pattern remains above the coverage threshold in the positive set;
6. Calculation of motif significance (Fisher’s exact test) and redundancy removal (Hamming distance);
7. Generation of the positon weighted matrices and logo for each motif.

### Web-server usage

The SeAMotE server presents a submission page that allows the upload of nucleic acid sequences and selection of parameters. Default parameters (e.g. reference set, coverage threshold, etc.) are defined according to best settings estimated using cross-validation (section “Cross-validation on the CLIP-seq data” in Result and Discussion). However, most of the parameters can be modified by the user, which adds flexibility to the web-service. Detailed descriptions of the submission and output variables are provided in the on-line tutorial (see http://service.tartaglialab.com/static_files/shared/tutorial_seamote.html, tutorial sections “Submission form” and “Interpreting the output”, respectively).

1. At least one input set (FASTA format file) should be provided for the analysis. Currently, the number of sequences is limited to 10^4^, with a maximal length of 15 ·10^3^ nucleotides per sequence;
2. A reference set is required to estimate the significance of the discovered motifs. This can be:
  ● Provided by the user (FASTA format file), having the same size restrictions as the input set.
  ● Automatically generated as a shuffle set, where the foreground set composition (i.e., individual nucleotide alphabet frequencies) and dimensions (i.e., number of sequences and lengths) are kept constant;
  ● Automatically generated as a random set, where the foreground set dimensions are preserved but the internal composition is based on letter frequencies obtained from the human transcriptome/genome;
3. The coverage threshold (i.e. the percentage of sequences matching the searched pattern) represents a parameter that the algorithm uses internally to select the most abundant motifs in the two datasets (speed of calculation increases at low coverage threshold).

Optionally, the user can assign a job name for each submission and request for an e-mail notification upon completion (not required to run the server).

**Figure 2:**
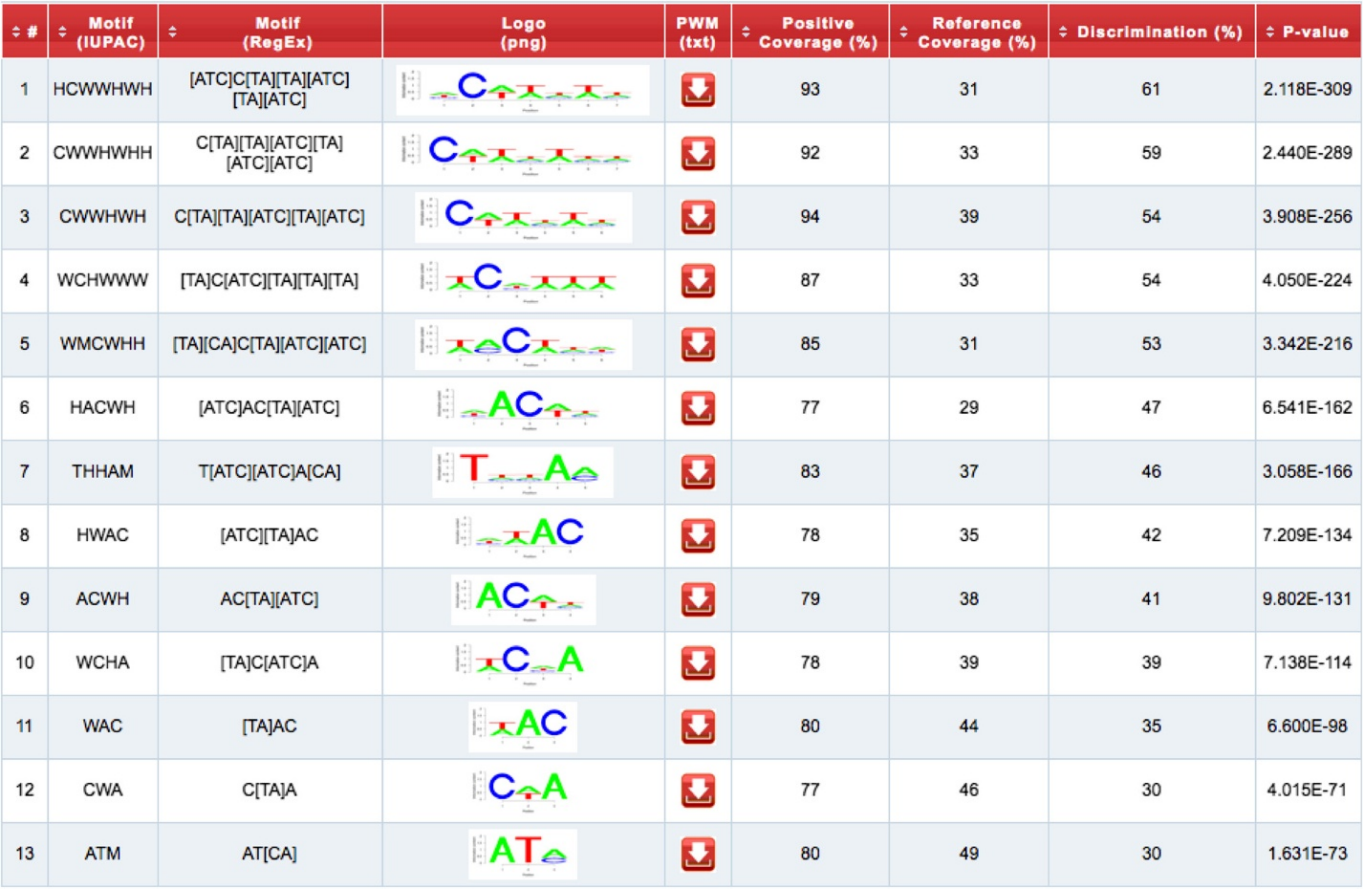
**SeAMotE output summary.** Example of output table showing the list of motifs (IUPAC and RegEx) that better discriminate the input sets along with their logo representation and positional weighted matrix download button, positive and reference coverage (as percentage of sequences containing at least one pattern occurrence), discrimination (Youden’s index) and p-value (Fisher’s exact test). By clicking on the logo, it is possible to retrieve the image file (png format) of the associated motif.

The output summary contains detailed information about the submission (e.g., job identifier, downloadable datasets) as well as an interactive table (Figure 2). The latter item displays discovered motifs (IUPAC and RE formats), logo representations and statistics used to estimate their significance: motif coverage for positive and reference sets, discrimination factor (Youden’s index) and p-value (Fisher’s exact test) associated with each pattern. In addition, it is possible to retrieve the list of motifs tested (txt format), as well as their individual sequence logo (png format) and positional weighted matrix (txt format) using the links provided in the output page (Figure 2).

## Implementation

SeAMotE operations are executed by C programs, whereas significance estimation, pattern filtering and sequence logo design are computed using R scripts and the Biostrings, stringdist and seqLogo packages. The web-server is implemented in Python, HTML and JavaScript, which provides a convenient framework for the pipeline control and the presentation of the output data. User-provided data are validated by Python scripts and passed to the Amazon Web Services (AWS), which manages the queue system, performs the redistribution of the work on our local machines and, once the job is completed successfully, forwards the user to the output page. Depending on the size datasets and the server load, computations take from between 2–3 and 30–40 minutes (Additional file 1: Figure S1).

### Datasets for motif finding

Nucleic acids sequences were collected from ChIP-seq and CLIP-seq experiments available in the public domain [18, 19]. ChIP-seq data comprises 351 ENCODE datasets obtained from three groups, Haib_Tfbs by HudsonAlpha (141 sets), Sydh_Tfbs by Yale and UCD (164 sets), and Uw_Tfbs by University of Washington (46 sets). This collection covers 90 unique transcription factors (TFs) and more than 50 cell-types under different treatments. Same number of low and high intensity peaks (1000 sequences) was used to select negative and positive datasets, respectively [20]. CLIP-seq dataset contains 13 doRINA [18] datasets of 10 RNA-binding proteins (RBPs) [21–28]. Sequences with doRINA scores in the top 5 percentile were considered as positives (*bound* transcripts; more details on the definition of peaks and the calculation of associated scores can be found in doRINA paper [18]). For each positive set, we selected same amount of sequences in the bottom 5 percentile of doRINA scores to build the negative set (*unbound* transcripts).

### Documentation

The documentation/tutorial of the SeAMotE algorithm is available online, and it can be accessed using the links in the menu at the top of every server page. It contains a brief description of the method, a tutorial and information on the benchmark. Additionally, the web interface in the output page provides help-notes (accessible also through the “mouse-over” function) for table variables and download buttons. Online documentation and “Frequently Asked Questions” (FAQs) sections updates will be provided on a regular basis according to method improvements and users’ inquiries,respectively.

## Results and discussion

### Identification of TF annotated motifs

**Figure 3:**
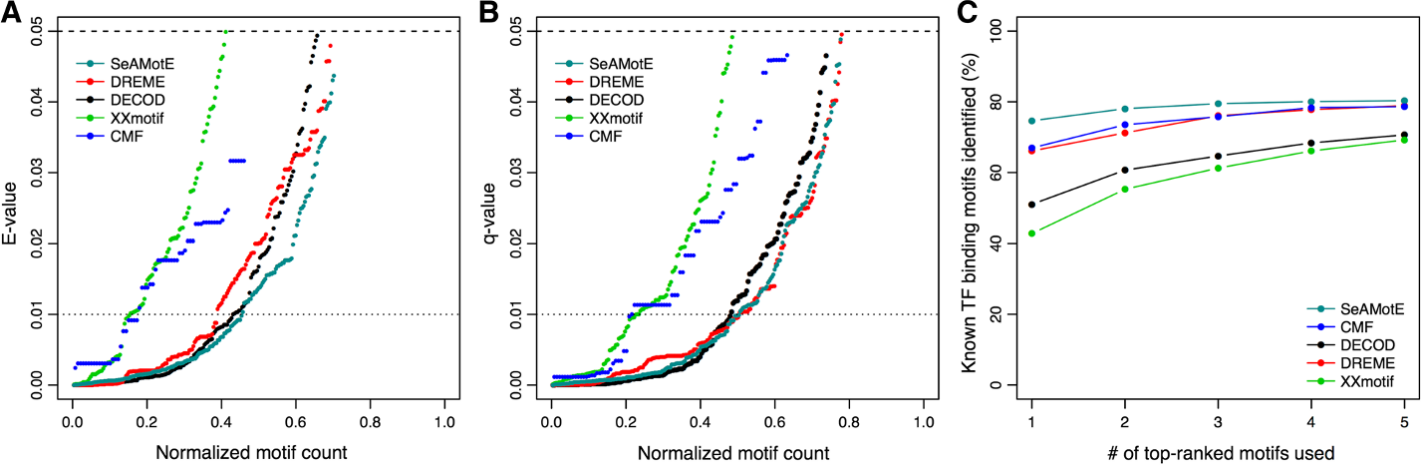
**Annotated motifs performance comparison.** Using 351 ChIP-seq datasets from ENCODE [19], we compared CMF [12], DECOD [13], DREME [11], XXmotif [14] and SeAMotE performances; **A)** E-values and **B)** q-values associated with the 5 top-ranked motifs for CMF, DECOD, DREME, SeAMotE and XXmotif. **C)** Proportion of transcription factors for which annotated motifs were succesfully identified is plotted against the number of top-ranked motifs employed for the TOMTOM search [31].

To assess the performance of our method for discriminative motif discovery, we run SeAMotE on a collection of 351 ChIP-seq datasets and compared our results with those obtained using other discriminative motif finders. Specifically, we restricted the comparison to methods such as CMF, DECOD, DREME and XXmotif that can be run *in batch* on large sets of sequences. All methods were run on the same sets of foreground and background sequences under default settings. For each algorithm, we selected up to 5 top-scored motifs. To evaluate the ability of the different methods to recognise sequence patterns reported in literature, we collected TF motifs present in Jaspar [29] and Jolma *et al.* [30]. We then compared the PWMs generated by CMF, DECOD, DREME, SeAMotE and XXmotif with the motifs available in the reference databases. We considered successful the prediction in which the annotated motif was reported as TOMTOM [31] match. Figures 3A and 3B report the E-value (i.e., the p-value multiplied by twice the number of target motifs) and q-value (the minimal false discovery rate at which the observed similarity would be considered significant) obtained from the analyses with TOMTOM. As shown in Figure 3C and Table 1, SeAMotE succeeded in finding the consensus motifs in 282 (80.3%) cases out of the 351 ChIP datasets with annotated motifs for the TFs. CMF found annotated motifs in 276 (78.6%), DECOD in 248 (70.6%), DREME in 277 (78.9%) and XXmotif in 243 (69.2%) cases (Figure 1C and Table 1). SeAMotE was able to identify annotated motifs in 74.6% of the cases even when considering only the top-ranked PWM (other methods recognized <67% of experimental cases; Figure 1C and Table 1).

**Table 1:** **Comparison of discriminative motif finder methods**

	Top-ranked motifs				
Method	1	2	3	4	5
CMF	235 (67%)	258 (74%)	266 (76%)	275 (78%)	276 (79%)
DECOD	179 (51%)	213 (61%)	227 (65%)	240 (68%)	248 (71%)
DREME	232 (66%)	250 (71%)	267 (76%)	273 (78%)	277 (79%)
SeAMotE	262 (75%)	274 (78%)	279 (79%)	281 (80%)	282 (80%)
XXmotif	150 (43%)	194 (55%)	215 (61%)	232 (66%)	243 (69%)

For each algorithm, performances using TOMTOM [31] and 1 to 5 top-ranked motifs are reported as number of successes (cases where the annotated motif is correctly identified) and as percentage over the complete ChIP-seq collection (351 experimental datasets [19]).

In 69 out of 351 cases (i.e. 20% of the dataset), SeAMotE identified motifs that are different from those reported in Jaspar[29] and Jolma *et al.* [30] databases. CMF and DREME identified different patterns in 74% and 67% of such cases (i.e., 51 out of 69 and 46 out of 69, respectively), which suggests that this group of TFs might display diverse binding modes. Indeed, with respect to the 282 successful hits, these motifs are predicted with significantly lower discrimination (p-value = 5.88*e*^−5^; Mann-Whitney U test on discrimination). Thus, it is possible that the discrepancy with literature data arises from lower sequence specificity of the TFs, which makes the foreground and background sets more similar and, therefore, less informative. It should be also mentioned that the 69 misassigned cases correspond to 42 TFs, and for 28 of them(66.7%) SeAMotE was able to correctly recognise the annotated binding pattern in at least one cell-type or specific treatment [19]. We also observe that some of the unassigned patterns can be correctly attributed to literature motifs if other comparison tools are employed instead of TOMTOM. In an additional calculation, we used Matlign [32] to compare the similarity between literature patterns and the top-ranked motif identified by SeAMotE. In 36 out of 69 cases, we found that SeAMotE motifs have higher propensity to cluster with those of the same TF family [29, 30]. Intriguingly, we observe that in 54 out of the 69 cases (78.3%) the top-ranked motif is associated with one PWM of an interacting TF, indicating that TF binding could be mediated by other proteins.

### Identification of RBP recognition motifs

**Figure 4:**
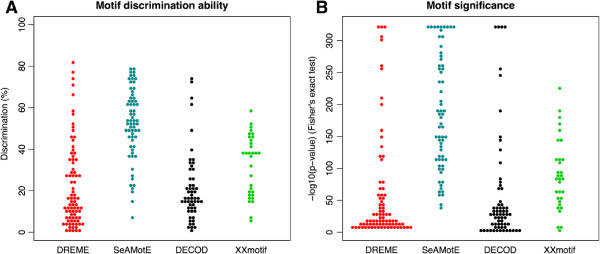
**RNA-binding protein motifs performance comparison.** Using 13 CLIP-seq experiments available in the public domain [18], we compared DECOD [13], DREME [11], XXmotif [14] and SeAMotE performances. The ability to identify sequence elements that maximize the separation between positive and reference sets is reported for each motif identified using **A)** discrimination (Youden’s index) and **B)** significance (FisherŠs exact test). CMF [12] was excluded from the analysis because it does not allow motif discovery on a nucleic acid specific strand.

To demonstrate the flexibility of our method for different types of nucleic acids, we assessed SeAMotE ability to identify significantly enriched motifs in transcripts studied by CLIP-seq technology [33]. In each case analysed, we compared RNAs bound to a specific protein (foreground set) with same amount of unbound transcripts (background set). Since CMF does not allow the discriminative motif discovery on specific nucleic acid strand, we excluded the algorithm from the study and used the other tools for comparison. In our analysis (Figure 3C) we noticed that SeAMotE and DREME show best performances in finding discriminative motifs, followed by DECOD and XXmotif. This result was confirmed also in the analysis of RBP targets (Figure 3C). Indeed, SeAMotE and DREME outperform DECOD and XXmotif in finding sequence patterns (Figure 4A). Compared to DREME, SeAMotE achieves significantly higher discrimination (p-value = 1.36*e*^−14^; Mann-Whitney U test), which is reflected in the ability to better separate foreground from background sets (Figure 4A), and significance, denoted by lower p-values associated with each sequence pattern identified (Figure 4B). In addition, SeAMoTe also shows very high sensitivity (∼89%) and accuracy (∼81%) (Table 2). Statistical measures of the performance are also reported for DECOD and XXmotif (Additional file 2: Table S1; p-value for SeAMotE - DECOD comparison: 3.95*e*^−16^; p-value for SeAMotE - XXmotif comparison: 1.09*e*^−07^; Mann-Whitney U test).

**Table 2:** **Comparison of DREME** [11] **and SeAMotE**

	SeAMotE				DREME					
Protein	TPR	SPC	PPV	FDR	ACC	TPR	SPC	PPV	FDR	ACC
ELAVL1 (Hafner)	85.3	77.7	79.3	20.7	81.5	83.0	73.2	75.6	24.4	78.1
ELAVL1 (Lebedeva)	80.3	74.9	76.2	23.8	77.6	75.6	74.5	73.3	26.7	75.0
ELAVL1 (Mnase)	86.6	77.8	77.9	22.1	82.2	86.3	71.6	75.2	24.8	79.0
ELAVL1 (Mukharjee)	92.2	82.8	84.3	15.7	87.5	89.5	81.7	83.1	16.9	85.6
FUS	93.2	68.3	74.6	25.4	80.8	92.2	45.3	62.8	37.2	68.8
IGF2BP1-3	92.5	27.5	56.0	44.0	60.0	92.5	27.5	56.0	44.0	60.0
PUM2	91.8	87.5	88.0	12.0	89.6	84.9	92.4	91.8	8.2	88.7
QKI	91.8	87.5	88.0	12.0	89.6	88.4	84.9	85.4	14.6	86.6
SFSR1	86.5	79.6	80.7	19.3	83.0	86.5	79.6	80.7	19.3	83.0
TAF15	95.4	68.4	75.1	24.9	81.9	91.0	54.9	66.9	33.1	73.0
TARDBP (iCLIP)	88.9	89.4	89.3	10.7	89.1	87.9	93.8	93.5	6.5	90.9
TIA1 (iCLIP)	86.7	62.3	70.4	29.6	74.5	86.7	62.3	70.4	29.6	74.5
TIAL1 (iCLIP)	85.5	66.4	72.0	28.0	75.9	84.4	66.2	71.7	28.3	75.3
TOTAL	88.6	73.3	78.0	22.0	80.9	86.8	69.8	75.9	24.1	78.3

Sensitivity (True Positive Rate, TPR), specificity (SPC), precision (Positive Predictive Value, PPV), false discovery rate (FDR) and accuracy (ACC) achieved by the two methods on the CLIP-seq experimental datasets [18].

### Cross-validation of the CLIP-seq data

Finally, we assessed SeAMotE performances using a 3-fold cross-validation approach introduced by Patel and Stormo [34]: CLIP-seq sets of positive and negative sequences were randomly divided into three sets of similar sizes (P1, P2, P3) and (N1, N2, N3); two of the three were combined to form a training set and the remaining one was used as test set. By this means, three training (TR1, TR2 and TR3) and three test sets (TE1, TE2 and TE3) were generated. We then compared the most significant motifs found in the training with those present in the test set using TOMTOM [31] (p-value <0.01). SeAMotE was able to correctly reproduce the most enriched motifs using training and testing sets, thus confirming the robustness of our approach (Additional file 3: Table S2).

## Conclusions

Algorithms for discriminative motif discovery are useful to identify regulatory elements in DNA and RNA sequences. Comparisons between different sets provide relevant information to rationalize sequence determinants of physical interactions and can be exploited for future experimental design. In this work, we introduced the SeAMotE algorithm for analysis of large-scale nucleic acid datasets. Through an easy-to-use interface, the SeAMotE web-server offers key features such as fast discrimination based on pattern occurrence, choice of multiple reference backgrounds (shuffle, random or custom) and identification of significant motifs in the whole span of tested pattern widths, which provides a range of practical solutions for analysis of experimental data. Indeed, as reported in recent studies, inter-positional sequence patterns and variable binding sites information are key features to identify regulatory motifs and will be used in future computational developments [35]. We demonstrated the powerfulness of SeAMotE for a large number of TF targets, correctly reproducing the results available in literature and showing better performances than other available tools. We also proved the flexibility and robustness of the algorithm by assessing its ability to identify enriched sequence patterns in CLIP experiments and using a three-fold cross-validation. We anticipate that the use of SeAMotE and its integration into DNA/RNA-protein interaction predictors, such as *cat*RAPID [36, 37], would greatly enhance the ability to recognise physical associations.

## Availability and requirements

- ● **Project name:** SeAMotE
- ● **Project home page:**http://s.tartaglialab.com/new_submission/seamote
- ● **Operating system(s):** Platform independent
- ● **Programming language:** C and R scripts
- ● **Other requirements:** Web browser (e.g. Safari, Firefox, Explorer or Chrome)
- ● **Restrictions:** No login requirement; users behind a proxy might experience slow-down issues

## Electronic supplementary material

Additional file 1: **Figure S1.** Motif discovery time performance. Motif discovery runtimes of CMF [12], DECOD [13], DREME [11], XXmotif [14] and SeAMotE algorithms are plotted for each ChIP-seq data set [19]. The cumulative distribution function represents the percentage of annotated TF motifs that are recovered using the corresponding method. (PNG 369 KB)

Additional file 2: **Table S1.** DECOD [13] and XXmotifs [14] statistical measures. Sensitivity (True Positive Rate, TPR), specificity (SPC), precision (Positive Predictive Value, PPV), false discovery rate (FDR) and accuracy (ACC) achieved by the two methods on the CLIP-seq experimental datasets. Cases in which XXmotif was not able to find any motif in the range of 3-7-mers are indicate with NA. (PNG 535 KB)

Additional file 3: **Table S2.** Cross-validation on RBPs. The table shows the 3-fold cross-validation performance of the SeAMotE approach on the CLIP data sets [18]. Training sets (TR1, TR2, TR3) are composed by two positive and two negative subsets, while the training sets (TE1, TE2, TE3) are represented by the positive and negative subsets that have not been used in the training. Datasets size, motifs identified along with their matches and coverage in the positive sets are reported for both training and testing analyses. The P-value associated with each training-testing pair of motifs, as calculated with TOMTOM [31], is shown in the last column. (PNG 954 KB)
