# Supplementary figures and images for: SeAMotE: a method for high-throughput motif discovery in nucleic acid sequences
Source: BMC Genomics. 2014 Oct 23;15(1):925. doi: 10.1186/1471-2164-15-925 (PMC4223730; doi:10.1186/1471-2164-15-925)

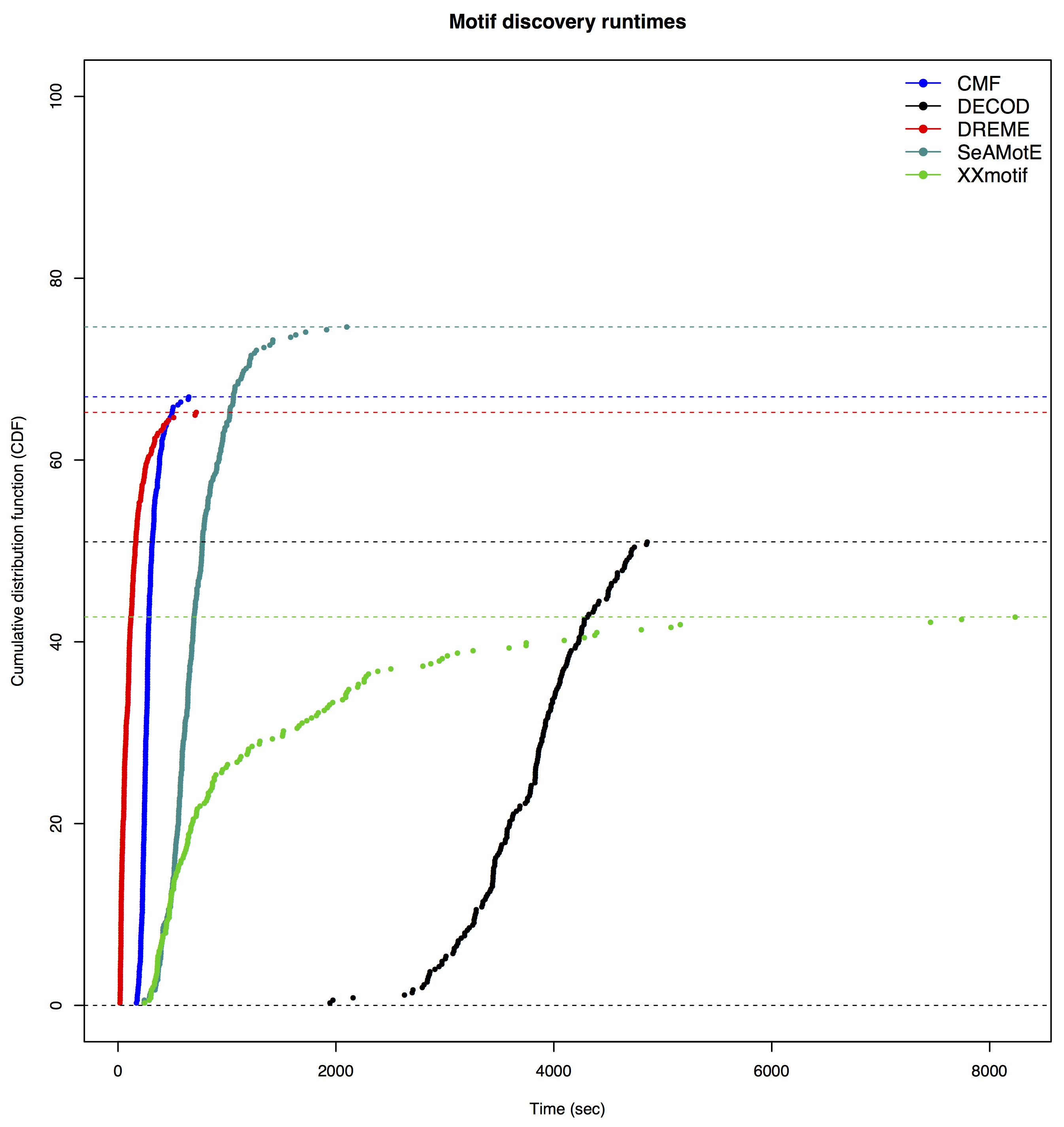

Supplement: Supplementary file 1 — Additional file 1: Figure S1. Motif discovery time performance. Motif discovery runtimes of CMF [12], DECOD [13], DREME [11], XXmotif [14] and SeAMotE algorithms are plotted for each ChIP-seq data set [19]. The cumulative distribution function represents the percentage of annotated TF motifs that are recovered using the corresponding method. (PNG 369 KB) [file 12864_2014_6626_MOESM1_ESM.png]

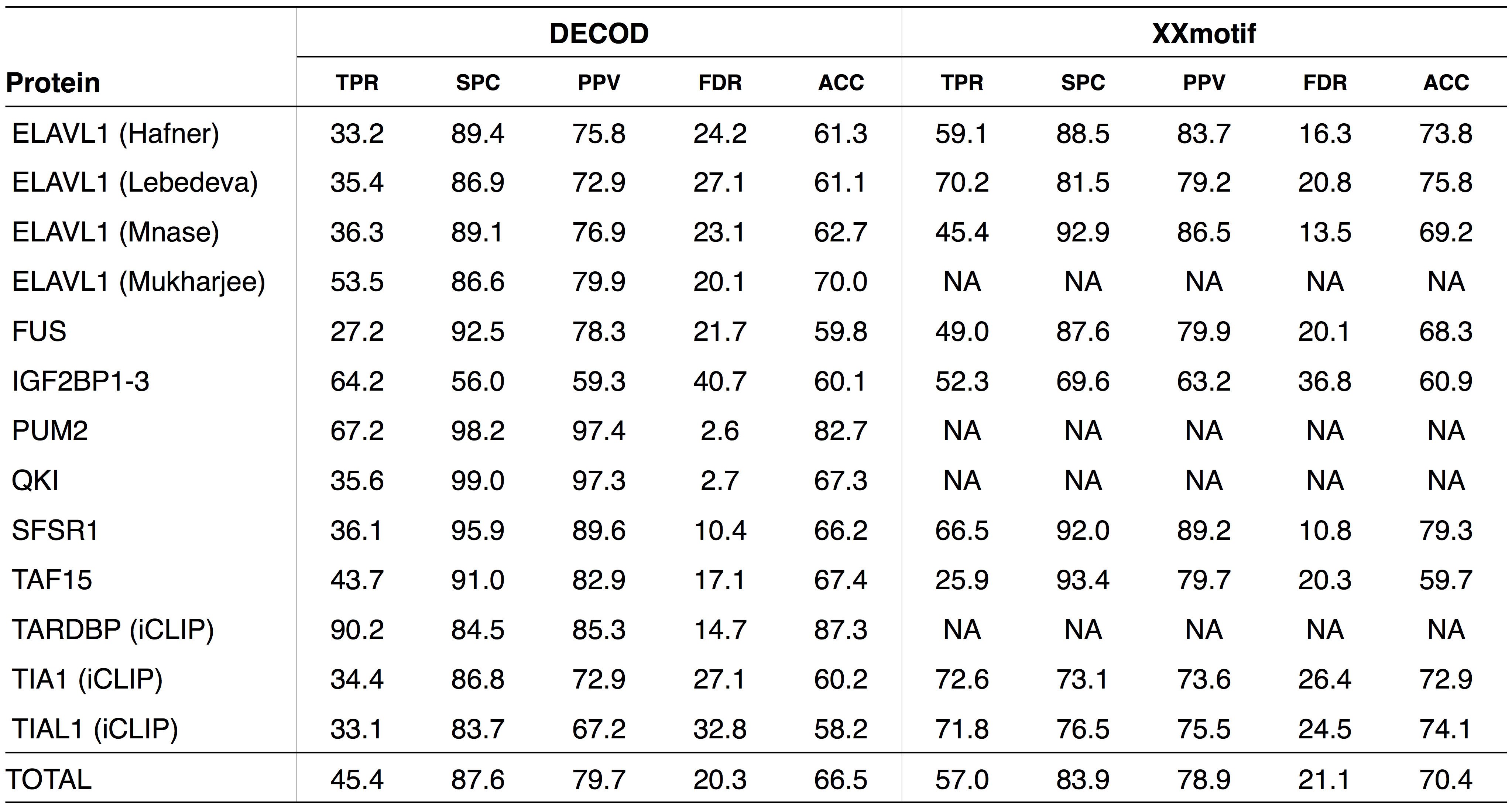

Supplement: Supplementary file 2 — Additional file 2: Table S1. DECOD [13] and XXmotifs [14] statistical measures. Sensitivity (True Positive Rate, TPR), specificity (SPC), precision (Positive Predictive Value, PPV), false discovery rate (FDR) and accuracy (ACC) achieved by the two methods on the CLIP-seq experimental datasets. Cases in which XXmotif was not able to find any motif in the range of 3-7-mers are indicate with NA. (PNG 535 KB) [file 12864_2014_6626_MOESM2_ESM.png]

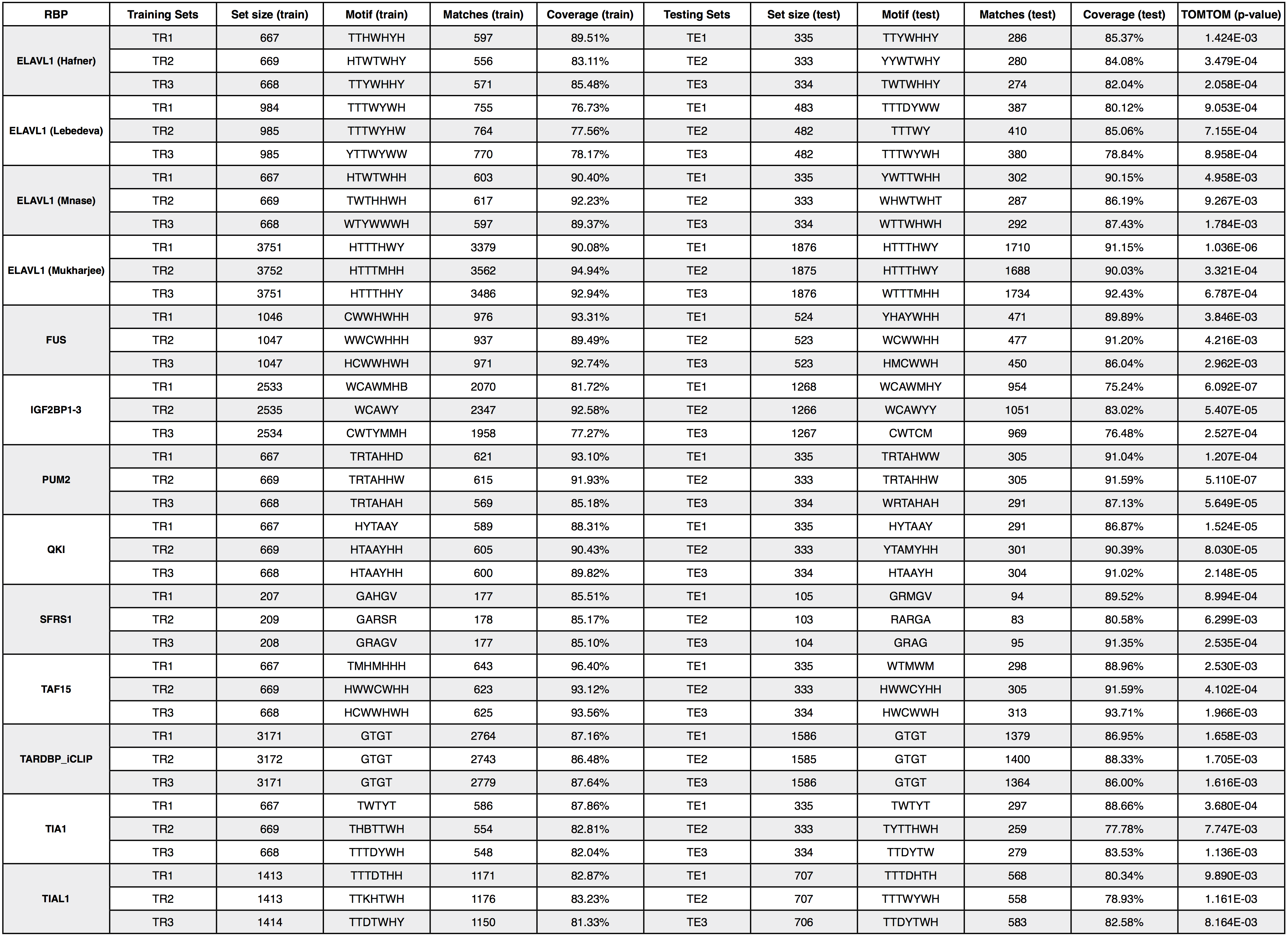

Supplement: Supplementary file 3 — Additional file 3: Table S2. Cross-validation on RBPs. The table shows the 3-fold cross-validation performance of the SeAMotE approach on the CLIP data sets [18]. Training sets (TR1, TR2, TR3) are composed by two positive and two negative subsets, while the training sets (TE1, TE2, TE3) are represented by the positive and negative subsets that have not been used in the training. Datasets size, motifs identified along with their matches and coverage in the positive sets are reported for both training and testing analyses. The P-value associated with each training-testing pair of motifs, as calculated with TOMTOM [31], is shown in the last column. (PNG 954 KB) [file 12864_2014_6626_MOESM3_ESM.png]
